# *Ex Vivo* and *In Vivo* Properties of an Injectable Hydrogel Derived From Acellular Ear Cartilage Extracellular Matrix

**DOI:** 10.3389/fbioe.2021.740635

**Published:** 2021-09-13

**Authors:** Danni Gong, Fei Yu, Meng Zhou, Wei Dong, Dan Yan, Siyi Zhang, Yan Yan, Huijing Wang, Yao Tan, Ying Chen, Bei Feng, Wei Fu, Yao Fu, Yang Lu

**Affiliations:** ^1^ Department of Ophthalmology, Ninth People’s Hospital, Shanghai Jiao Tong University School of Medicine, Shanghai, China; ^2^ Shanghai Key Laboratory of Orbital Diseases and Ocular Oncology, Shanghai, China; ^3^ Shanghai Children’s Medical Center, Department of Pediatric Cardiothoracic Surgery, Shanghai Jiao Tong University School of Medicine, Shanghai, China; ^4^ Shanghai Children’s Medical Center, Institute of Pediatric Translational Medicine, Shanghai Jiao Tong University School of Medicine, Shanghai, China

**Keywords:** cartilage, extracellular matrix, injectable hydrogel, tissue engineering, regenerative medicine

## Abstract

Extracellular matrix (ECM) hydrogels provide advantages such as injectability, the ability to fill an irregularly shaped space, and the adequate bioactivity of native matrix. In this study, we developed decellularized cartilage ECM (dcECM) hydrogels from porcine ears innovatively via the main method of enzymatic digestion and verified good biocompatible properties of dcECM hydrogels to deliver chondrocytes and form subcutaneous cartilage *in vivo*. The scanning electron microscopy and turbidimetric gelation kinetics were used to characterize the material properties and gelation kinetics of the dcECM hydrogels. Then we evaluated the biocompatibility of hydrogels via the culture of chondrocytes *in vitro*. To further explore the dcECM hydrogels *in vivo*, grafts made from the mixture of dcECM hydrogels and chondrocytes were injected subcutaneously in nude mice for the gross and histological analysis. The structural and gelation kinetics of the dcECM hydrogels altered according to the variation in the ECM concentrations. The 10 mg/ml dcECM hydrogels could support the adhesion and proliferation of chondrocytes *in vitro*. *In vivo*, at 4 weeks after transplantation, cartilage-like tissues were detected in all groups with positive staining of toluidine blue, Safranin O, and collagen II, indicating the good gelation of dcECM hydrogels. While with the increasing concentration, the tissue engineering cartilages formed by 10 mg/ml dcECM hydrogel grafts were superior in weights, volumes, collagen, and glycosaminoglycan (GAG) content compared to the dcECM hydrogels of 1 mg/ml and 5 mg/ml. At 8 weeks after grafting, dcECM hydrogel grafts at 10 mg/ml showed very similar qualities to the control, collagen I grafts. After 12 weeks of *in vivo* culture, the histological analysis indicated that 10 mg/ml dcECM hydrogel grafts were similar to the normal cartilage from pig ears, which was the source tissue. In conclusion, dcECM hydrogel showed the promising potential as a tissue engineering biomaterial to improve the regeneration and heal injuries of ear cartilage.

## Introduction

Cartilage is a flexible connective tissue composed of chondrocytes trapped in extracellular matrix (ECM). The absence of vascularization and limited proliferation of mature chondrocytes induces poor self-healing capacity of cartilage tissues. Therefore, the cartilage damage is irreversible and increases the risk for the long-term development of some diseases, such as osteoarthritis (OA) (Hunziker, 1999; Vinatier and Guicheux, 2016). A number of current clinical treatments has been used to improve the cartilage repair, including autologous chondrocyte implantation, subchondral abrasion, microfracture, and transplantation of osteochondral plugs, albeit with limited success, especially for large, and irregular defects (Simon and Jackson, 2018; Chimutengwende-Gordon et al., 2020). In order to optimize the functional restoration, tissue-engineered cartilage is a promising alternative for repair.

In tissue engineering strategies, the scaffold is the “soil” of seed cells, whose primary objective is to simulate the properties of the target-tissue ECM. Then, the ECM is mimicked to produce natural and synthetic biomaterials that can support cell viability and functions with respect to cartilage tissue engineering *in vitro* and/or *in vivo* (Hunziker, 1999; Vinatier et al., 2009). The potential advantages of these hydrogels are biocompatibility, cell-controlled degradability, and intrinsic cellular interaction (Nooeaid et al., 2012; Bao et al., 2020). Typically, natural hydrogels are nominated as successful candidates in cartilage tissue engineering based on their preferable biocompatibility, safety, and stability that support growth, proliferation, and differentiation of chondrocytes and the regeneration of cartilage tissues (Spiller et al., 2011; Amini and Nair, 2012; Wang et al., 2018).

Natural hydrogels, especially ECM hydrogels, provide magnificent bioactivity and natural adhesive surface for cells (Bao et al., 2020). In native cartilage tissues, ECM plays a crucial role in regulating chondrocytes’ behavior and maintaining the functions of tissues. The cartilage ECM is primarily composed of collagen II and some other molecules, such as proteoglycans, glycosaminoglycan (GAG), and growth factors, which can define the microenvironmental niche (Wu et al., 2010; Gao et al., 2014). Therefore, how to retain these molecules in hydrogel scaffolds is yet to be elucidated in cartilage tissue engineering.

Recently, several studies have reported that the ECM of decellularized tissues can be solubilized in pepsin and subsequently polymerized into hydrogels under physiological conditions (Lu et al., 2015; Wu et al., 2015; Pouliot et al., 2020). And these ECM hydrogels remain a part of the biologically active molecules found in native tissues, showing significant therapeutic potentials in remodeling source tissues after implantation (Young et al., 2011; Wolf et al., 2012; Medberry et al., 2013; Sawkins et al., 2013). In addition, hydrogels derived from decellularized cartilage ECM (dcECM) are highly desirable in cartilage tissue engineering owing to their advantages. First, dcECM hydrogels allow access to surgically inaccessible trauma sites via the non-invasive injection. Second, dcECM hydrogels can flow into irregularly shaped defects and integrate with the surrounding native tissues. Third, the preparation of dcECM hydrogels could maximize retain the low-molecular-weight peptides and growth factors present in the native ECM (Wu et al., 2010; Ma et al., 2013; Gao et al., 2014). So far, dcECM hydrogels have been found potentials to promote the stable chondrogenesis and cartilage repair (Schwarz et al., 2012; Liu et al., 2017). However, there is no standard protocol for the manufacture of dcECM hydrogels, which can be improved more and the biocompatible properties of dcECM hydrogels to deliver cells and form cartilage also need to be further studied.

In this study, we developed dcECM hydrogels mainly via enzymatic digestion, which was easy and could retain ECM well. We also characterized the material properties, gelation kinetics, and *in vitro*/*vivo* biocompatibility of dcECM hydrogels. The findings demonstrated the feasibility of injectable dcECM hydrogels and provided a promising candidate to facilitate constructive remodeling in cartilage injuries, especially the repair of ear cartilage tissues.

## Materials and Methods

### Ethic Statement and Experimental Animals

Experiments were performed under a project license (HKDL2018377) granted by the Medical Ethics Committee of Shanghai Ninth People’s Hospital, Shanghai Jiao Tong University School of Medicine, in compliance with Chinese national or institutional guidelines for the care and use of animals.

Six four-month-old male Chinese white pigs, weighing approximately 110 kg, were purchased from Shanghai Chuansha Experimental Animal Raising Farm, Shanghai, China. Fifteen eight-week-old male BALB/c-nude mice were provided by the Animal Laboratory, Shanghai Ninth People’s Hospital, Shanghai Jiao Tong University School of Medicine, Shanghai, China.

### Preparation of Cartilage Sheets and Cartilage Decellularization

Cartilage tissues were harvested from adult pig ears and cut into circular cylinders with a diameter of 6 mm. Then, the cartilage sheets were obtained by freeze-sectioning at a thickness of 10 μm, followed by rinsing with 1% (wt/vol) sodium dodecyl sulfate (SDS, Sigma-Aldrich, St. Louis, MO, United States) in phosphate-buffered saline (PBS, HyClone, GE Healthcare, Little Chalfont, United Kingdom) for 1 day to remove cells (Young et al., 2011). Subsequently, the decellularized cartilage was rinsed with deionized water overnight to remove the detergent. For histological analyses, hematoxylin and eosin (HE) and 4,6-diamidino-2-phenylindole (DAPI, Biomol, Plymouth Meeting, United States) staining of the cartilage sheets were performed before and after decellularization. Then, cartilage sheets were lyophilized and milled into a fine powder for preparing hydrogels.

### Generation of the Decellularized Cartilage Extracellular Matrix (dcECM) Hydrogels

To liquefy the dcECM, the milled powder was resolubilized as described previously (Young et al., 2011; Fu et al., 2019). Briefly, 1 mg/ml porcine pepsin (Sigma-Aldrich, St. Louis, MO, United States) in 0.01 N HCl was used to digest the comminuted dcECM enzymatically under a constant stir rate for 24 h at room temperature. The pepsin digested dcECM stock solutions at 15 mg ECM/mL (dry wt.) were cryopreserved for subsequent experiments. Gelation of the stock solution was induced by neutralizing the pH with one-tenth the digest volume of 0.1 N NaOH, balancing the salt concentration of the pepsin digest with one-ninth the digest volume of 10 × PBS. Then, 1 × PBS was added at 4°C to obtain the desired dcECM concentration. The mixture was placed in a non-humidified incubator at 37°C for 30 min to form 1, 5, and 10 mg/ml dcECM hydrogels spontaneously.

### Scanning Electron Microscopy

To examine the surface morphology of dcECM hydrogels, SEM (JEOL 6380LV, Japan) was used as described previously (Xue et al., 2013). Briefly, dcECM hydrogels at concentrations of 1, 5, and 10 mg/ml were fixed in cold 2.5% glutaraldehyde for 24 h, rinsed in PBS, dehydrated using graded ethanol, and placed in 100% ethanol for 12 h at 4°C. Finally, the hydrogels were air-dried and sputter-coated with gold before imaging. The morphology of the specimens was photographed using SEM. The diameters of at least 100 fibers of each sample from different SEM images were measured and analyzed using Image J 1.50i software (National Institutes of Health, Bethesda, MD, United States).

### Turbidimetric Gelation Kinetics

The gelation kinetics of the cartilage dcECM hydrogels were evaluated turbidimetrically and compared between groups, as described previously (Young et al., 2011; Wolf et al., 2012). Briefly, 1, 5, and 10 mg/ml dcECM hydrogels were plated in a 96-well plate (100 μl/well) at 4°C. The plate was read using a spectrophotometer (Biotek Devices, Vermont, United States) and the absorbance was measured at 405 nm every 2 min for 1 h. Then the readings were scaled from 0 (at time 0) to 100% (at the maximum absorbance) to determine the normalized absorbance (NA) according to Equation (Hunziker, 1999). A is the absorbance at a given time, A_0_ is the initial absorbance, and A_max_ is the maximum absorbance. The time required to reach 50 and 95% of A_max_ is defined as t_50_ and t_95_, separately, and the gelation rate (S) represents the slope of the linear region of the gelation curve.

$$\text{NA}=\left({\text{A}-\text{A}}_{\text{0}}\right)\text{/}\left({\text{A}}_{\text{max}}-{\text{A}}_{\text{0}}\right)$$ (1)

### Isolation and Culture of Chondrocytes

The porcine chondrocytes were isolated and cultured, as described previously (Yan et al., 2009). Briefly, fresh cartilage tissue from one pig ear was cut into 2 × 2 mm^2^ slices and digested with 0.25% trypsin plus 0.02% EDTA (Sigma-Aldrich, St. Louis, MO, United States) at 37°C for 30 min. Then, the slices were digested with 0.1% collagenase II (Sigma-Aldrich, St. Louis, MO, United States) in serum-free Dulbecco’s-modified Eagles medium (DMEM, HyClone, GE Healthcare, Little Chalfont, United Kingdom) at 37°C for an additional 12–16 h. The chondrocytes were counted and seeded in dishes at a cell density of 2 × 10^4^/cm^2^ in DMEM with 10% fetal bovine serum (FBS, HyClone, GE Healthcare, Little Chalfont, United Kingdom). The cells at passage 1 and 2 were used for further experiments. Chondrocytes from different pigs were applied for repetitive experiments.

### *In Vitro* Cell Culture and Viability Assay

To determine the biocompatibility of dcECM hydrogels *in vitro*, we chose collagen I derived from rat tail (Col-I, BD Biosciences, San Jose, CA, United States) as the control, which gelatinized stably under the concentration of 1 mg/ml as reported (Fu et al., 2019). Chondrocytes were seeded in 24-well plates, coated with collagen I or 10 mg/ml dcECM hydrogels at a density of 2 × 10^4^ cells/well, and viable chondrocytes were imaged and quantified using the Cell Counting Kit-8 (CCK-8, Dojindo, Kumamoto, Japan) Assay. According to the manufacturer’s instructions, at 1, 3, 5, 7, 9, and 11 days after seeding, the cells were washed with PBS and incubated with 10% CCK-8 in DMEM for 2 h. Then, the absorbance of each well was measured at 450 nm using a microplate reader (ELX800, BioTek, Vermont, United States). The cell proliferation assay was presented by the mean optical density (OD) value from six wells, and experiments were repeated three times by using chondrocytes from different pigs.

### *In Vivo* dcECM Hydrogels Transplantation and Subcutaneous Chondrogenesis Assay

To determine the biocompatibility and chondrogenesis supporting abilities of dcECM hydrogels *in vivo*, a subcutaneous transplantation model in nude mice was constructed, as described previously (Sawkins et al., 2013). Mice were separately kept in colony room with a 12-h light/dark cycle at 25 °C for 7 days before initiating experiments. Briefly, 350 μl collagen I (Col-I, 1 mg/ml) or the dcECM hydrogel at 1, 5, or 10 mg/ml was mixed with 50 μl chondrocytes (5 × 10^6^ cells in 50 μl PBS), respectively, to prepare Col-I grafts or dcECM grafts. Subsequently, this mixture was injected subcutaneously into the dorsal region of mice via 25 G needles at different points, the injection dose of the mixture at each injection point was 400 μl. Ten mice were randomly selected and each mouse received four plugs, which were from the different groups above. At 4 or 8 weeks after transplantation, five mice were randomly selected and sacrificed for harvesting the implants. In addition, five mice separately received one plug from the 10 mg/ml dcECM group, which would be harvested at 12 weeks after transplantation. The implants were used for gross evaluation and further analyses (*n* = 5/each time point). The weights and volumes were recorded and compared as reported previously (Xue et al., 2013).

### Quantification of Collagen and Glycosaminoglycan (GAG) Contents

Grafts were digested in papain solution (Sigma-Aldrich, St. Louis, MO, United States ) and assayed for soluble, triple-helical collagen content via the Sircol Collagen Assay (Biocolor Ltd., Carrickfergus, United Kingdom). To determine the GAG content, 1,9-dimethylmethylene blue dye solution (Sigma-Aldrich, St. Louis, MO, United States) was used according to the instruction of the kit. A pepsin buffer solution was used as the negative control and subtracted from the signal, as described previously (Young et al., 2011).

### Histological Analyses

The implants harvested at investigated time points and normal cartilage tissue of pig ears were collected for further histological staining as described previously (Liu et al., 2008). Briefly, samples were fixed in 4% paraformaldehyde, paraffin-embedded, and sliced into 5-μm-thick sections. HE staining was performed to analyze the structure of implants. Toluidine blue and Safranin O staining were used to evaluate GAG deposition in the engineered cartilage tissues. Collagen II expression of pig chondrocytes was specifically detected by a mouse anti-human collagen II antibody (1:200; Abcam, Cambridge, MA, United States) and horseradish peroxidase-conjugated anti-mouse secondary antibody (1:50; Dako, Denmark). The sections were developed using diaminobenzidine tetrahydrochloride (DAB, Dako, Denmark), and images were acquired by microscope (Olympus BX51, Japan) and at least six representative fields from each sample were examined.

### Statistical Analysis

Data were expressed as the mean ± SD. The statistical analysis was performed with one-way analysis of variance test (ANOVA) for comparisons across multiple groups, followed by post hoc analysis Tukey test in GraphPad Prism 8.0 (GraphPad, San Diego, CA, United States). *p* < 0.05 was considered statistically significant.

## Results

### Preparation of dcECM Hydrogels Derived From Cartilage ECM

**FIGURE 1:**
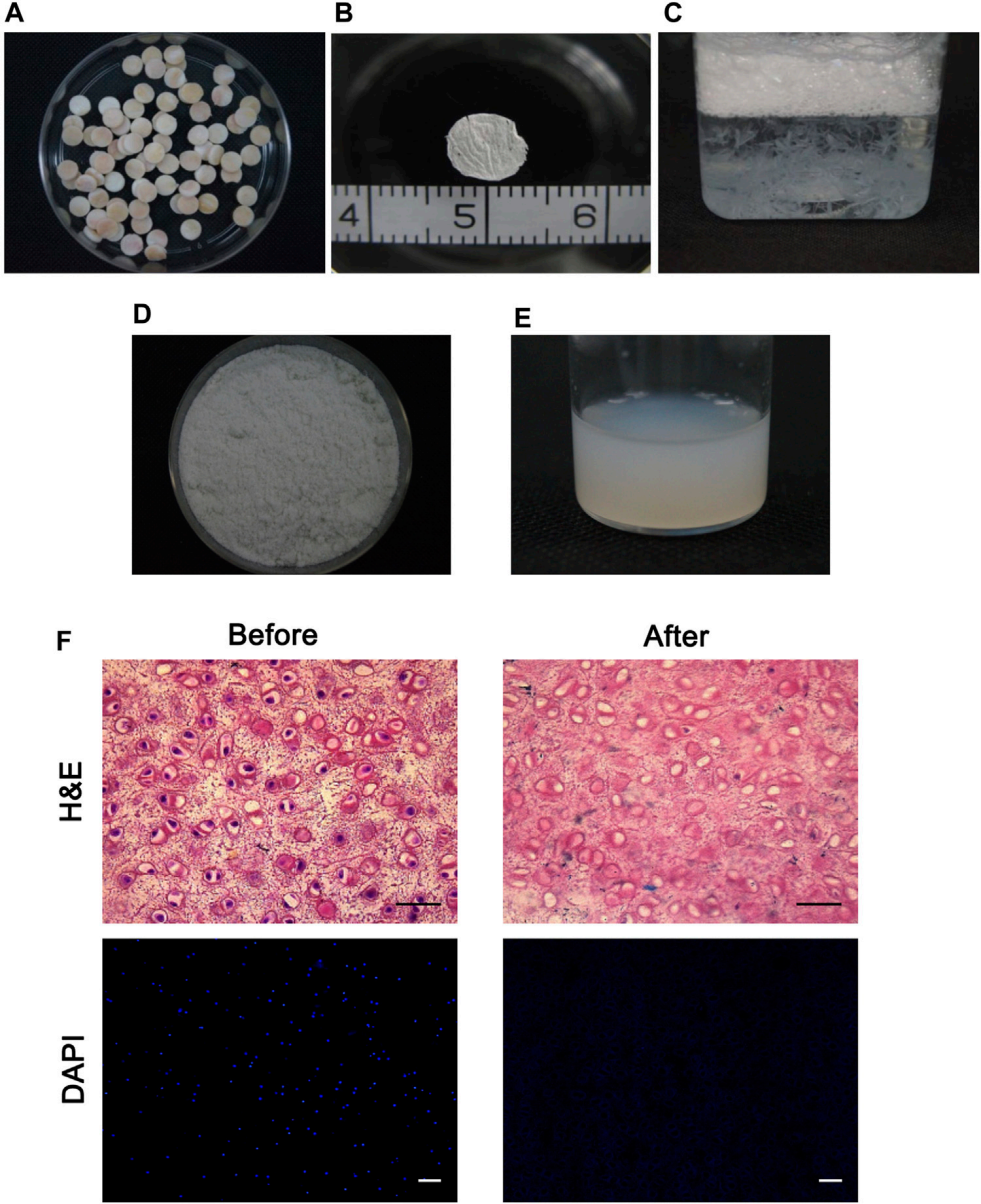
Production of the decellularized cartilage extracellular matrix. **(A)** Macroscopic images of cylindrical cartilage slices. **(B)** 10-µm-thick cartilage sheets were observed. **(C)** Cartilage sheets were decellularized. **(D, E)** Decellularized cartilage sheets were lyophilized, milled into a fine, white powder **(D)**, and solubilized using pepsin and HCl **(E)**. **(F)** Histological analyses of the cartilage slices before and after decellularization confirmed the absence of nuclei via HE and DAPI staining. Scale bars = 50 µm.

The diameter of cartilage cylinders was 6 mm (Figure 1A) and the 10 µm thick cartilage sheets were sectioned and rinsed (Figures 1B,C). Following the decellularization protocol, the ECM fine powder was made (Figure 1D) and solubilized with pepsin to liquefy the cartilage matrix. This soluble matrix displayed properties similar to those of purified collagen gels, facilitating it to be a viscous liquid at 4°C and polymerizing after incubation at 37°C (Figure 1E). The histological analyses of sheets revealed that the cartilage ECM remained intact after decellularization and almost all nuclei were absent by DAPI staining (Figure 1F).

### Macroscopic Appearance and Surface Ultrastructure of dcECM Hydrogels

**FIGURE 2:**
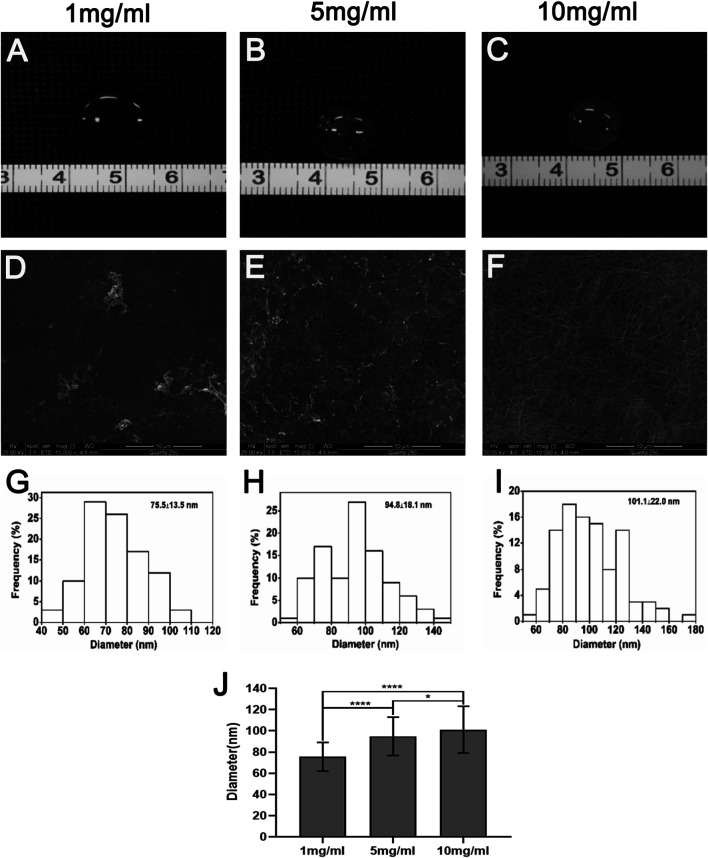
Macroscopic appearance, scanning electron microscopy images and mechanical properties of dcECM hydrogels *in vitro*. **(A–C)** Macroscopic appearance of the dcECM hydrogels at **(A)** 1, **(B)** 5, and **(C)** 10 mg/ml. **(D–F)** Scanning electron microscopy micrographs of the dcECM hydrogels at **(D)** 1, **(E)** 5, and **(F)** 10 mg/ml (10,000×). **(G–I)** Distributions of fiber diameters of **(G)** 1, **(H)** 5, and **(I)** 10 mg/ml dcECM hydrogels. **(J)** Comparisons of the fiber diameters of the dcECM hydrogels at different concentrations. Error bars showed means ± SD, *p < 0.05, ****p < 0.0001, *n* = 100.

Hydrogels were successfully prepared from dcECM scaffolds at the concentrations of 1, 5, and 10 mg/ml. Qualitatively, the hydrogels of higher ECM concentrations (5 and 10 mg/ml) were more viscous than the 1 mg/ml (Figures 2A–C). The SEM images of the hydrogel surface showed randomly oriented fibrillar structures with interconnecting pores in dcECM hydrogels at different concentrations (Figures 2D–F). The dcECM hydrogels at 10 mg/ml contained the thickest fibrils and the highest fibril density as compared to the hydrogels at lower concentrations (Figures 2D–F). The fiber diameter increased non-linearly from 75.5 ± 13.5 nm at 1 mg/ml to 101.1 ± 22.0 nm at 10 mg/ml (Figures 2G–J).

### Turbidimetric Gelation Kinetics of dcECM Hydrogels

**FIGURE 3:**
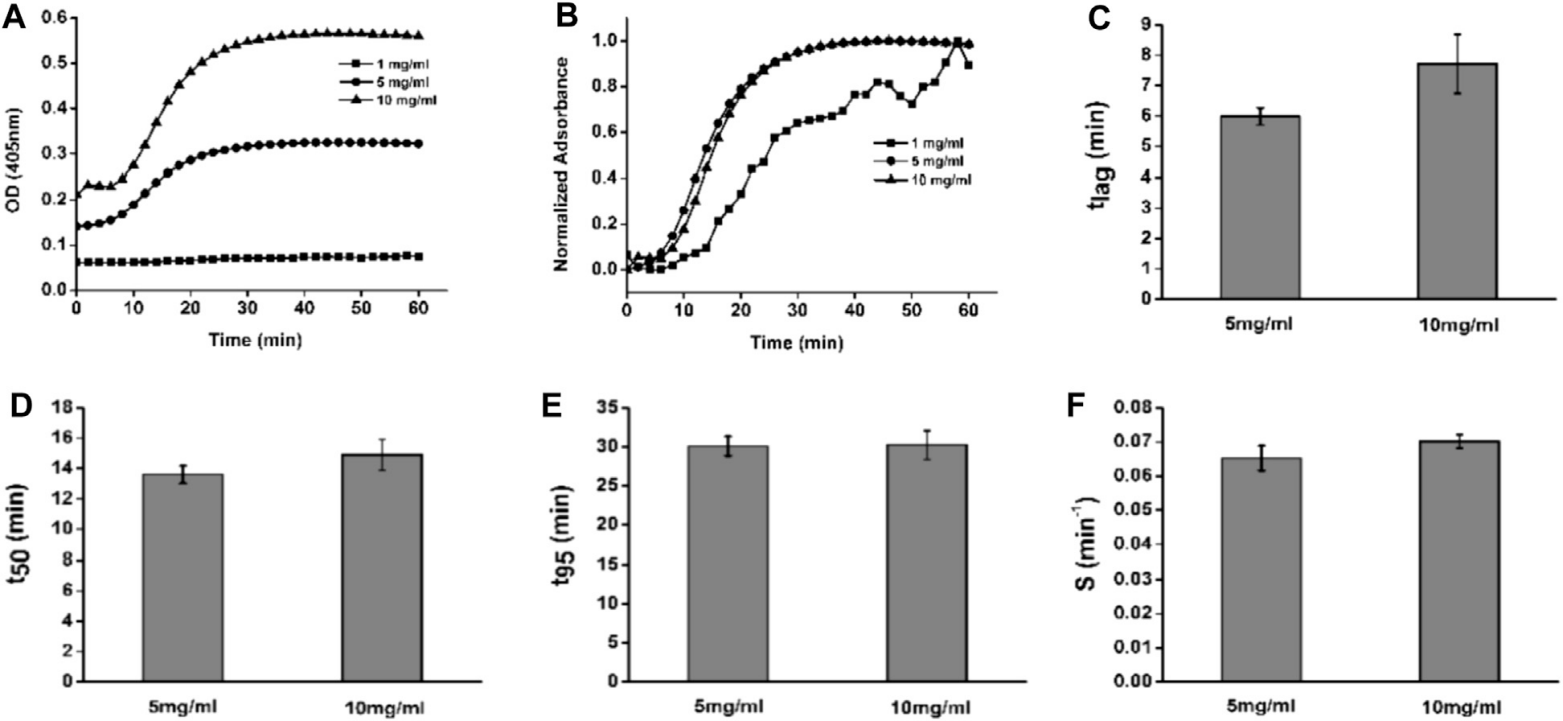
Representative turbidimetric and normalized turbidimetric curves of dcECM hydrogels. **(A, B)** Turbidimetric **(A)** and normalized turbidimetric gelation kinetics **(B)** of dcECM hydrogels at 1, 5, and 10 mg/ml. **(C–F)** Comparisons of lag time **(C)**, time to 50% gelation **(D)**, time to 95% gelation **(E)**, and speed to complete gelation **(F)** for 5 and 10 mg/ml dcECM hydrogels. Error bars showed means ± SD, *n* = 6.

To explore the properties of dcECM hydrogels, the gelation kinetics of the hydrogels at 1, 5, and 10 mg/ml were evaluated. The no change turbidity of 1 mg/ml indicated this concentration dcECM hydrogels could not cross-link well (Figure 3A). The turbidimetric gelation kinetics for 5 and 10 mg/ml showed sigmoidal shapes (Figures 3A,B). The lag phase for dcECM hydrogels at 5 mg/ml (6.153 ± 0.324) has no statistical significance with that at 10 mg/ml (7.876 ± 1.051) (Figure 3C). In addition, the times to reach 50 and 95% gelation, as well as the gelation rate (S) are also similar for dcECM hydrogels at 5 mg/ml and at 10 mg/ml (Figures 3D–F). The results indicated that the turbidimetric gelation kinetics for dcECM hydrogels at 5 mg/ml and 10 mg/ml are both good.

### dcECM Hydrogel Coatings Support *In Vitro* Chondrocytes Culture

**FIGURE 4:**
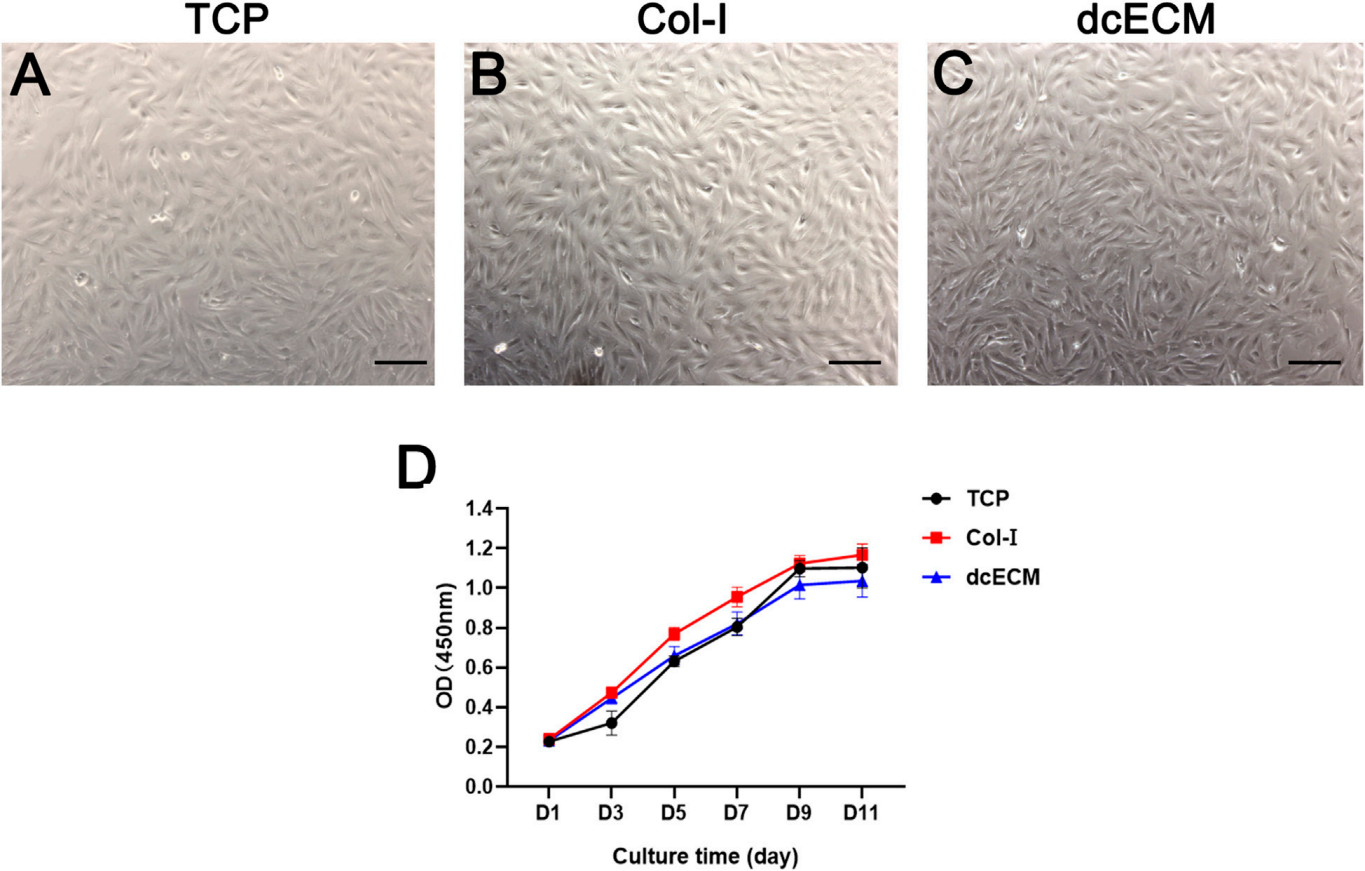
Viability of chondrocytes on TCP, collagen I, and dcECM hydrogels. **(A-C)** Viability of chondrocytes cultured on TCP **(A)**, collagen I (Col-I) **(B)**, and 10 mg/ml dcECM hydrogels (dcECM) **(C)**-coated plates on day 7 **(D)** The proliferation capability of chondrocytes on TCP, Col-I, and dcECM at 1, 3, 5, 7, 9, and 11 days after cell seeding was assessed using CCK-8 kit. Error bars showed means ± SD, *n* = 6. Scale bars = 100 µm.

To evaluate the effects of dcECM on the viability of cells, chondrocytes were cultured on uncoated plates (TCP), Col-I-coated plates and 10 mg/ml dcECM hydrogel-coated plates (dcECM). The images of chondrocytes after 7 days of culture indicated that dcECM hydrogel coating could support the adhesion and proliferation of chondrocytes (Figures 4A–C). Furthermore, the proliferation of chondrocytes at day 1, 3, 5, 7, 9, and 11 after seeding on different coating plates was compared and shown in Figure 4D. The viability of chondrocytes on dcECM-coated plates was similar to those grown on uncoated TCP plates and Col-I-coated plates from day 3 to day 11, indicating dcECM hydrogels could support cells well.

### dcECM Hydrogels *In Vivo* Support Chondrocytes for Cartilage Formation

**FIGURE 5:**
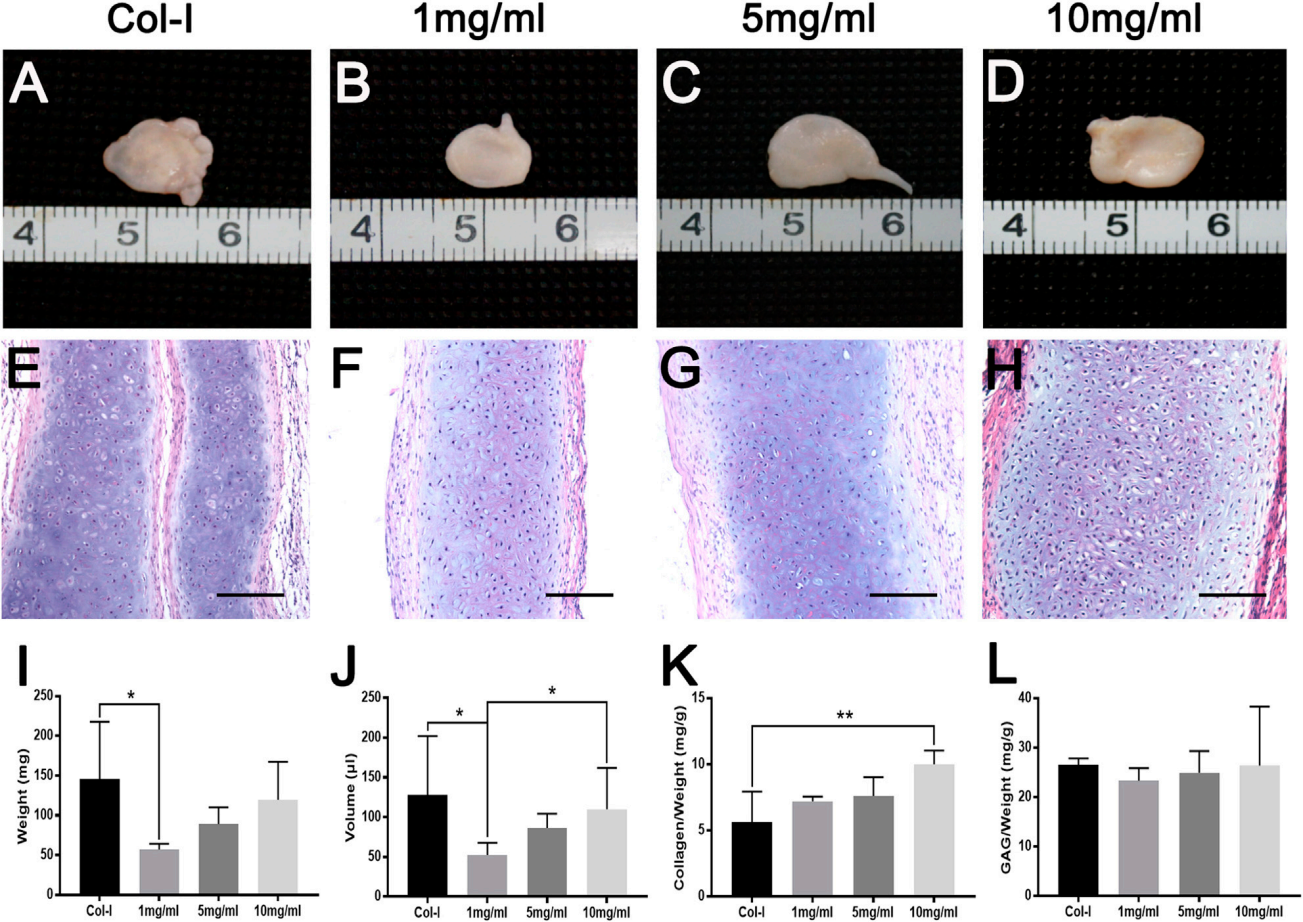
Macroscopic appearance, HE staining and gross analyses of dcECM hydrogel grafts *in vivo* at 4 weeks after grafting. Grafts were harvested and compared at 4 weeks after transplantation. **(A–H)** Macroscopic appearance and HE staining of grafts from collagen I (Col-I) **(A, E)** and dcECM hydrogels at 1 **(B, F)**, 5 **(C, G)**, and 10 **(D, H)** mg/ml. **(I, J)** Comparisons of weights **(I)** and volumes **(J)** of grafts after 4 weeks *in vivo* culture. **(K, L)** Collagen and GAG contents analyses of grafts after 4 weeks *in vivo* culture. Scale bars = 100 µm. Error bars showed means ± SD, *p < 0.05, **p < 0.01, *n* = 5.

**FIGURE 6:**
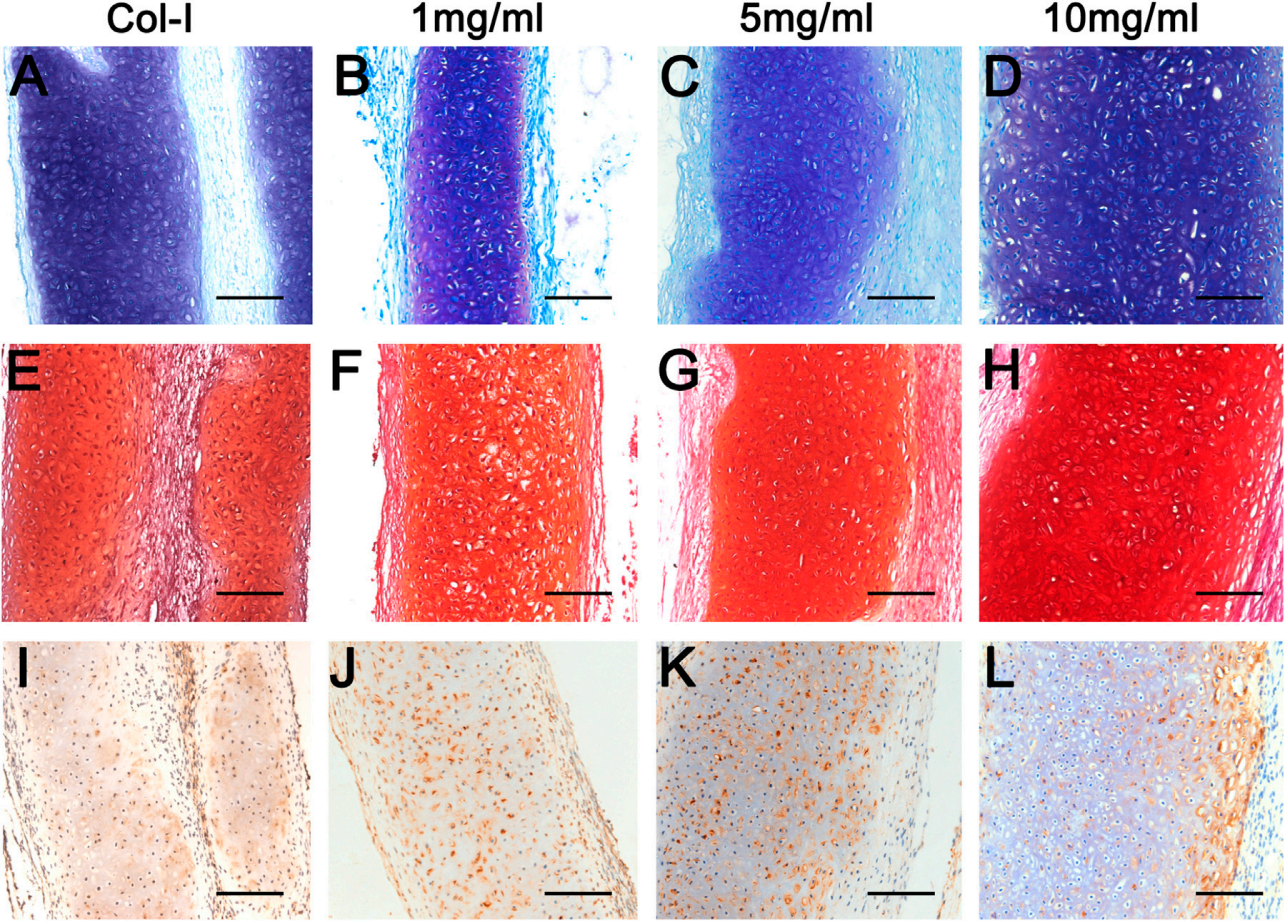
Histological analyses of dcECM hydrogels at 4 weeks after grafting. Grafts were harvested and compared at 4 weeks after transplantation. **(A–D)** Histological analyses of the Col-I **(A)**, 1 **(B)**, 5 **(C)**, and 10 **(D)** mg/ml dcECM hydrogel grafts by Toluidine blue. **(E–H)** Safranin O staining of grafts from Col-I **(E)**, 1 **(F)**, 5 **(G)**, and 10 **(H)** mg/ml dcECM hydrogels. **(I–L)** Representative images of collagen II staining of Col-I **(I)**, 1 **(J)**, 5 **(K)** and 10 **(L)** mg/ml dcECM hydrogel grafts. Scale bars = 100 μm.

To further explore the biological characteristics of dcECM hydrogels, chondrocytes mixed with Col-I or dcECM hydrogels at 1, 5, and 10 mg/ml were transplanted subcutaneously in nude mice. During the experimental period, we monitored the physical state and behavior of mice, and found that implants had no obvious adverse effects on mice. The representative macrography of grafts after 4 weeks post-grafting indicated that all concentrations of dcECM hydrogels had the same good gelation as collagen I in Figures 5A–D. The wet weights, volumes, collagen, and GAG contents of grafts increased gradually with increasing concentration of hydrogels (Figures 5I–L). Grafts of the 10 mg/ml group weighed similar to those of the Col-I group (Figure 5I) and the volumes of 10 mg/ml grafts were significantly larger than those of 1 mg/ml (Figure 5J) (*p* < 0.05). The collagen ratios of 10 mg/ml grafts were significantly higher as compared to those of the Col-I group (Figure 5K) (*p* < 0.01). However, the GAG assay did not show a marked difference between Col-I and dcECM hydrogel groups (Figure 5L). Furthermore, HE images indicated more viable chondrocytes and thicker tissues in the 10 mg/ml grafts in comparison to the other concentrations (Figures 5E–H). With increasing concentrations, the rate of positive Toluidine-blue and Safranin O staining tissue improved (Figures 6A–H); also collagen II immunohistochemistry indicated collagen deposition in the dcECM groups (Figures 6I–L). Taken together, the results put forth that dcECM hydrogels supported the viability of chondrocytes and the formation of lacunae *in vivo*, which induced the subcutaneous chondrogenesis.

**FIGURE 7:**
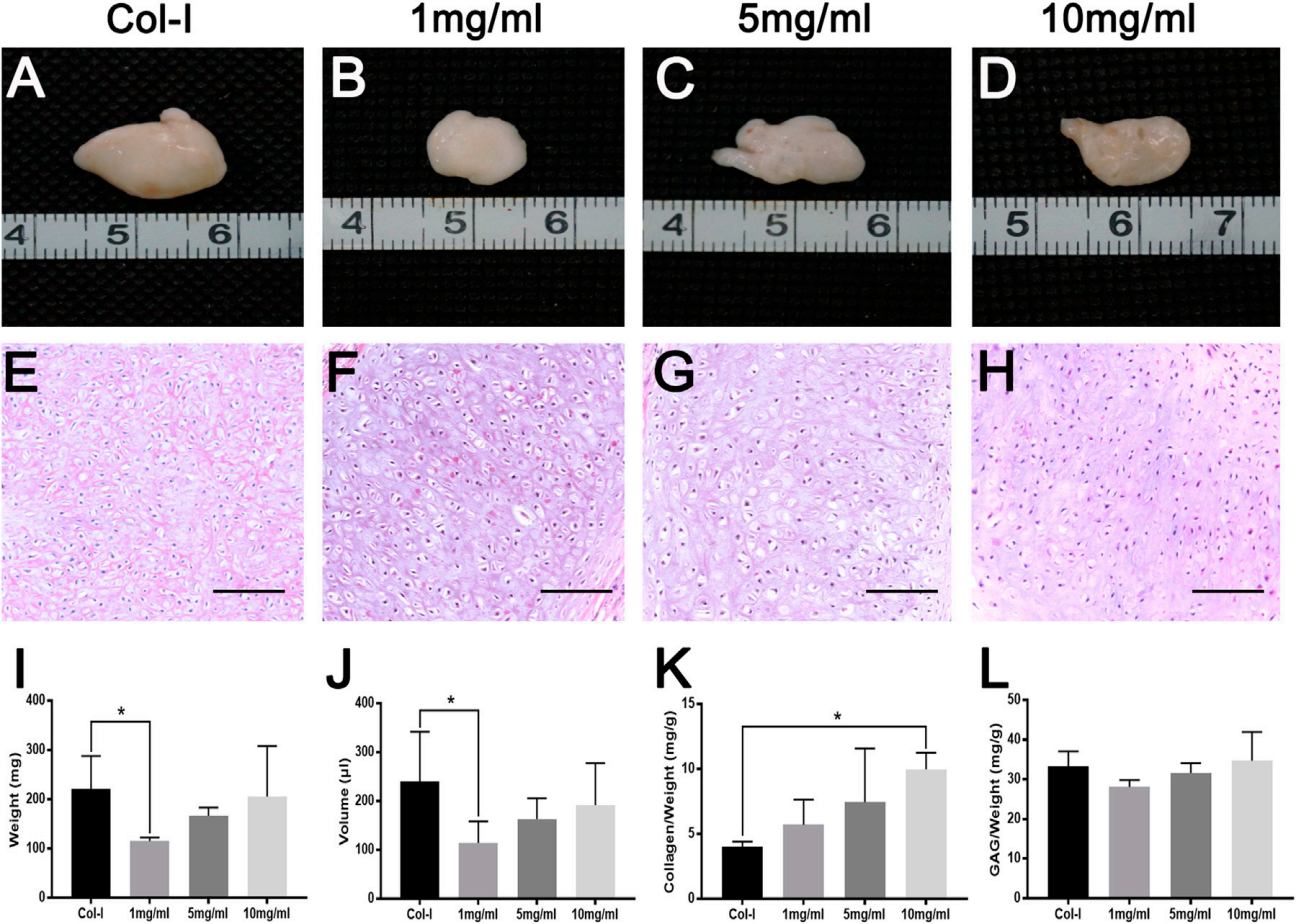
Macroscopic appearance, HE staining and gross measurements of dcECM hydrogel grafts after 8 weeks of *in vivo* transplantation. Grafts were harvested and compared at 8 weeks after transplantation. **(A–H)** Macroscopic appearance and HE staining of collagen I (Col-I) **(A, E)** and dcECM hydrogels at 1**(B, F)**, 5 **(C, G)**, and 10 **(D, H)** mg/ml grafts. **(I, J)** Gross analyses of weights **(I)** and volumes **(J)** from grafts. **(K, L)** Comparisons of collagen and GAG contents of grafts after 8 weeks of *in vivo* culture. Scale bars = 100 µm. Error bars showed means ± SD, *p < 0.05, *n* = 5.

**FIGURE 8:**
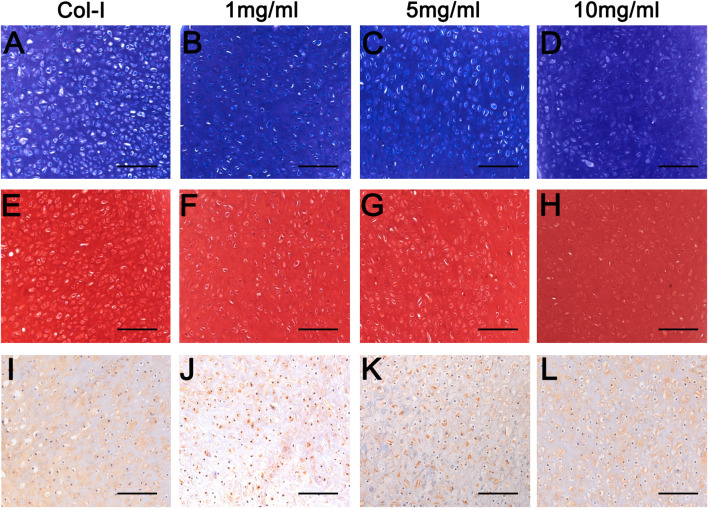
Histological analyses of grafts from dcECM hydrogels at 8 weeks after transplantation. Grafts were harvested and further analyzed at 8 weeks after grafting. **(A–D)** Toluidine-blue staining of the Col-I **(A)**, 1 **(B)**, 5 **(C)**, and 10 **(D)** mg/ml dcECM hydrogel grafts. **(E–H)** Safranin O staining of grafts from Col-I **(E)**, 1 **(F)**, 5 **(G)** and 10 **(H)** mg/ml dcECM hydrogels. **(I–L)** Collagen II expression of Col-I **(I)**, 1 **(J)**, 5 **(K)**, and 10 **(L)** mg/ml dcECM hydrogel grafts were compared via histological staining. Scale bars = 100 μm.

To further explore the longer performances of dcECM hydrogels *in vivo*, grafts were analyzed at 8 weeks after transplantation (*n* = 5/each time point). Macroscopic appearances of grafts in Figures 7A–D showed that 10 mg/ml dcECM hydrogel grafts seemed bigger and more compact when compared with the 1 or 5 mg/ml group. Similarly, with the increasing concentrations of dcECM hydrogels, the wet weights, volumes and ratios of collagen and GAG contents of implants presented a rising trend via the 8-week culture *in vivo* (Figures 7I–L). The weights and volumes of 1 mg/ml grafts were significantly lower than those of Col-I grafts (*p* < 0.05), while the sizes of 10 mg/ml implants were similar to those of the Col-I group (Figures 7I,J). After 8 weeks, 10 mg/ml dcECM hydrogels could retain maximal collagen and GAG among the groups (Figures 7K,L). Further analyses of HE images showed that the dcECM hydrogels supported the survival of chondrocytes as well as collagen I (Figures 7E–H). Also, Toluidine-blue and Safranin O staining displayed the biological properties of dcECM hydrogels that improved the formation of cartilage-like tissues (Figures 8A–H). The expression of collagen II in dcECM hydrogel grafts was similar to that of grafts with collagen I (Figures 8I–L). Therefore, dcECM hydrogels could be deemed to perform adequately to maintain the long-term survival and function of chondrocytes *in vivo*.

**FIGURE 9:**
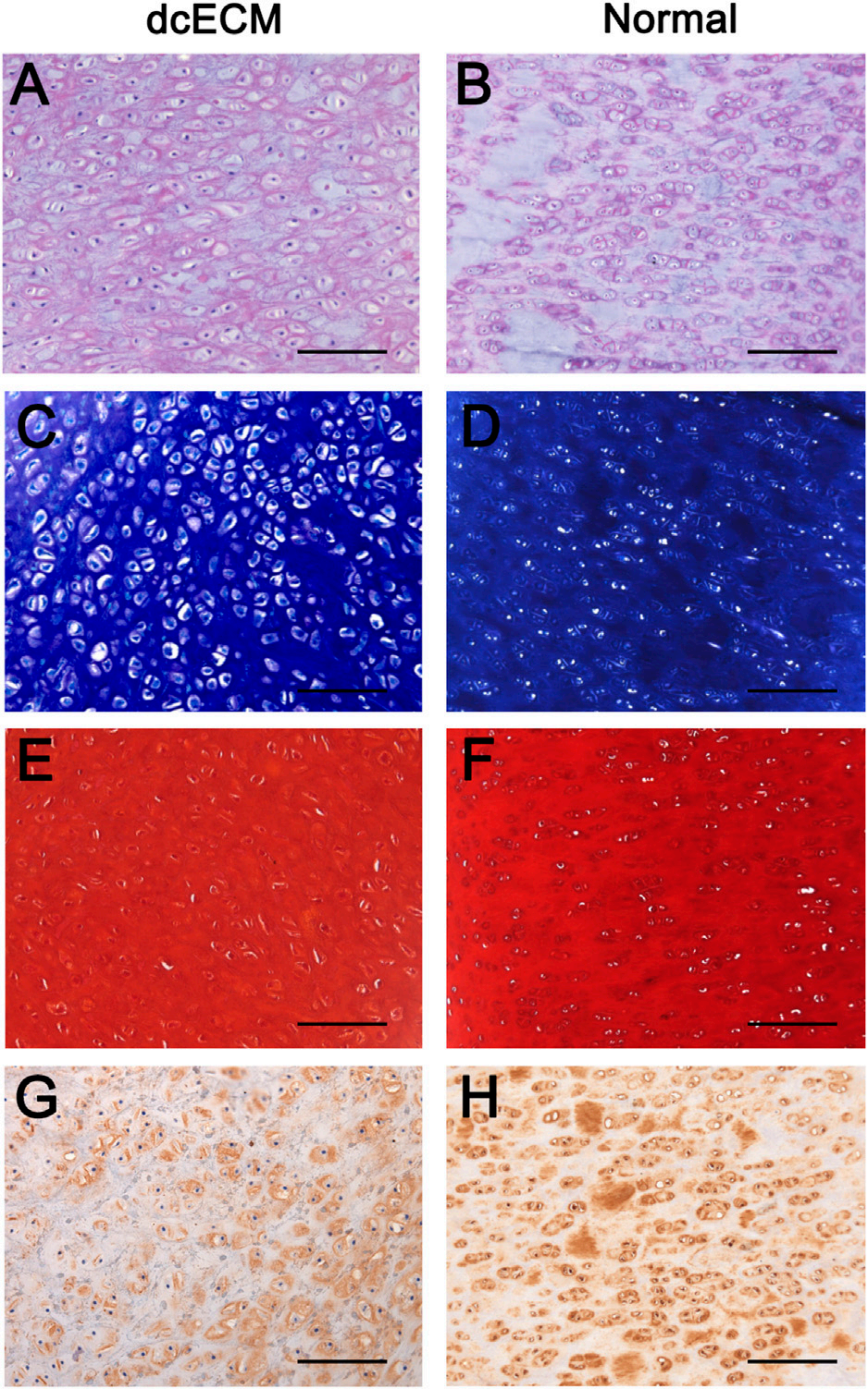
Histological comparisons between grafts from dcECM hydrogels at 12 weeks after transplantation and normal ear cartilage tissue. Grafts of 10 mg/ml dcECM hydrogels were harvested at 12 weeks after *in vivo* culture. **(A, B)** HE staining images of dcECM hydrogel grafts (dcECM) and normal cartilage tissues (Normal) from pig ears were compared. **(C, D)** Toluidine blue staining of samples from dcECM and Normal groups. **(E, F)** Safranin O staining of grafts from the dcECM group and tissues from the Normal group. **(G, H)** Comparison of collagen II expressions between grafts from the dcECM group and tissues from the Normal group. Scale bars = 100 μm.

Moreover, dcECM hydrogel grafts after 12 weeks of *in vivo* culture were compared to the original normal cartilage tissue of ears via immunohistochemistry analyses (Figure 9). HE staining showed similar numbers of chondrocytes and morphology of tissue between 10 mg/ml dcECM hydrogel grafts and source ear cartilage (Figures 9A,B). Toluidine blue, Safranin O, and collagen II staining showed chondrocytes in dcECM hydrogel were as rich as those in source tissue and apparent lacunae were also observed in the engineered cartilage tissue (Figures 9C–H). Therefore, the subcutaneous cartilage-like tissue formed by dcECM hydrogels was similar to the normal ear cartilage (Figures 9C–H).

## Discussion

Recently, hydrogels are widely used in cartilage tissue engineering as they exhibit properties similar to cartilage tissues and can present cells in a 3D environment for tissue formation and repair of defects (Spiller et al., 2011). Among these, hydrogels derived from naturally occurring ECM molecules have several potential advantages for therapeutic applications including robust bioactive substances to supply vital microenvironment, ease of delivery via injections to fill irregular and large defects, and no risk of immunologic rejection when applied during allografting (Brown et al., 2010; Ma et al., 2013; Park et al., 2013; Xue et al., 2013). Herein, we presented the fabrication of hydrogels from porcine decellularized cartilage ECM via a combined detergent and enzymatic method. The current results indicated that the fibrillar structures and gelation kinetics of the CM hydrogels altered according to the variation of hydrogel concentrations. dcECM hydrogels showed cytocompatibility and could support cell proliferation *in vitro*. Furthermore, with an increasing concentration of ECM, the contents of collagen II and sulfated proteoglycan of dcECM hydrogels implants increased gradually, whose biological properties supported the subcutaneous chondrogenesis well *in vivo*. Thus, dcECM hydrogels presented a promising potential for future application in the field of ear cartilage tissue engineering.

In compact tissues, native cells cannot be removed easily during the decellularization process. In this study, we sliced the cartilage tissues into 10-µm sections, following which almost all chondrocytes could be easily removed with a gentle and simple treatment (Figure 1) (Singelyn et al., 2012). The acellularization process should only affect the removal of immunogen, meanwhile retaining the biological activities and the gelation ability (Benders et al., 2013). So histological analyses were performed to verify the successful removal of cellular components (Figure 1F). Then dcECM hydrogels were made from the decellularized cartilage sheets. Although the specific mechanisms responsible for the gelation of dcECM hydrogels were unclear, the SEM images showed high collagen fiber content, which could explain the hydrogel formation (Figure 2) (Medberry et al., 2013). As reported, the collagen fibers, which are formed by the self-assembly of collagen monomers into fibrils, constitute the quaternary structure of collagen. (Kopesky et al., 2010; Mendes et al., 2013; Liu et al., 2019). The turbidity shown in Figure 3 indicated that both 5 mg/ml and 10 mg/ml dcECM hydrogels exhibited sigmoidal gelation kinetics. This phenomenon was consistent with the nucleation and growth mechanism, while the 10 mg/ml dcECM hydrogels reached a steady-state plateau faster than the 5 mg/ml dcECM hydrogels, thereby indicating that the gelation of dcECM hydrogels could be partially manipulated by regulating the ECM concentration.

Furthermore, pepsin-degraded and solubilized cartilage ECM is composed of large proteoglycans (PGs) and collagen, which preserves the normal phenotype of chondrocytes to promote the regeneration of cartilage-like constructs under the specific environment (Ko et al., 2009). To verify the bioactivities *in vitro*, we assessed the mitogenic capacity of chondrocytes upon dcECM hydrogels. dcECM group could induce a continuous proliferation rate of chondrocytes from day 1–11, which was similar to the Col-I or TCP group (Figure 4). Furthermore, the cells were found to be viable on dcECM scaffolds, rendering the dcECM hydrogels as non-cytotoxic.

For scaffolds of cartilage tissue engineering, ECM hydrogels exhibit several advantages, such as 3D networks for spherical cellular morphology, and the possibility to be tailored into an injectable gel (Benya and Shaffer, 1982; Cushing and Anseth, 2007; Spiller et al., 2011). In addition, ECM hydrogels can also preserve many natural bioactive components of source tissues that are vital to the viability and regeneration of target cells and tissues (Singelyn et al., 2009; DeQuach et al., 2012; Benders et al., 2013). Hitherto, many ECM hydrogels have been prepared successfully and show good biological properties *in vivo* (Freytes et al., 2008; Okada et al., 2010; Young et al., 2011; DeQuach et al., 2012; Singelyn et al., 2012; Fu et al., 2019). Our *in vivo* results also revealed that 1, 5, or 10 mg/ml dcECM hydrogels had good gelatinization and could assist chondrocytes forming cartilage-like tissues as tissue engineering scaffolds (Figures 5–8). Furthermore, with prolonged transplantation time, the increased weights and volumes of implants confirmed that dcECM hydrogels could not only supply 3D spaces to chondrocytes, but also form an essential microenvironment to verify the survival and secretory functions of chondrocytes *in vivo* (Figures 5–8). Moreover, similar lacunae and ECM components were detected in the tissue-engineered cartilage in comparison to the normal cartilage tissue at 12 weeks after grafting (Figure 9). Therefore, hydrogels from natural cartilage ECM are biocompatible, the preparation of dcECM hydrogels is reproducible and easy to implement, and the scaffolds can form into desired morphology and be delivered to the defects via injection. Future studies will investigate the application of this novel dcECM hydrogels to repair the ear cartilage defects in animal models.

The current results showed that dcECM hydrogels maintained the viability and functions of chondrocytes during *in vivo* transplantation (Figures 6, 8), while chondrocytes as “seeds” exhibited limited proliferation after maturity. Thus, alternatives for seed cells are worth exploring. Reportedly, collagen hydrogels support not only the adhesion, growth, and migration of mesenchymal stem cells but also the chondrogenesis for the engineered osteochondral structures (Pulkkinen et al., 2010; Ren et al., 2016; Wang et al., 2019). Therefore, dcECM hydrogels consisting of natural collagen could be applied in assisting stem cells to repair cartilage tissues. Moreover, hydrogels can specifically deliver both hydrophilic and hydrophobic drugs (Singelyn et al., 2009; Eslahi et al., 2016). Although dcECM hydrogels can provide a similar microenvironment of the target tissue, many biological substances, especially growth factors, are still not enough for massive and timely tissue repair (Fu et al., 2019). Accordingly, we can further enhance the bioactivities of dcECM hydrogels via adding factors, drugs or other biomaterials in future studies.

In conclusion, in the current study, decellularized dcECM could be successfully formed via the main digestion easily and solubilized to form injectable hydrogels. We verified dcECM hydrogels preserved the bioactivities of native ECM well to support the adhesion and proliferation of chondrocytes *in vitro* and *in vivo*. The results also showed that dcECM hydrogels had promising potential to be an alternative scaffold for ear cartilage regenerative medicine and tissue engineering.

## Data Availability

The original contributions presented in the study are included in the article/supplementary material, further inquiries can be directed to the corresponding authors.
